# *Clonorchis sinensis* excretory secretory products promote hepatic fibrosis through stimulating biliary epithelium to secrete IL-17A

**DOI:** 10.1371/journal.pntd.0014280

**Published:** 2026-05-05

**Authors:** Tongtong Liu, Hongna Gao, Nan Wang, Weichen Wang, Zhang Cao, Hao Zhu, Naiguo Liu, Honglian Gao, Bing Ji, Tonggang Liu, Fan Zhang, Xuelian Bai

**Affiliations:** 1 Department of Clinical Laboratory, Binzhou Medical University Hospital, Binzhou, Shandong Province, People′s Republic of China; 2 Medical Research Center, Binzhou Medical University Hospital, Binzhou, Shandong Province, People′s Republic of China; 3 Binzhou Polytechnic, Binzhou, Shandong Province, People′s Republic of China; 4 Department of Pathology, Binzhou Medical University Hospital, Binzhou, Shandong Province, People′s Republic of China; 5 Department of Infectious Diseases, Binzhou Medical University Hospital, Binzhou, Shandong Province, People′s Republic of China; 6 Department of Hepatobiliary Surgery, Clinical Nutrition Support Center, Binzhou Medical University Hospital, Binzhou, Shandong Province, People′s Republic of China; Xuzhou Medical University, CHINA

## Abstract

*Clonorchis sinensis* (*C. sinensis*) infection causes serious pathological changes of hepatobiliary system such as hyperplasia of the biliary mucosa, inflammation and periductal fibrosis. The excretory-secretory products of *C. sinensis* (CsESPs) play critical roles in triggering inflammation and subsequent activation of hepatic stellate cells (HSCs). Yet, how CsESPs induce hepatic fibrosis through breaking the barrier of biliary epithelium remains unclear. Previous studies have confirmed that interleukin-17A (IL-17A) promoted fibrosis in some liver diseases. In the present study, the IL-17A levels in the serum of *C. sinensis* infected patients and healthy people were compared. *C. sinensis* infected mouse model was applied to discover the expression of IL-17A, especially its localization in the biliary epithelium. Cells and bile duct organoid models were established to evaluate the effect of CsESPs on the production of IL-17A by biliary epithelium and subsequent activation of HSCs. The results indicated that the levels of IL-17A were higher in the serum of patients and mice infected with *C. sinensis* than in the healthy people and control mice respectively. Infected mouse liver showed increased collagen deposition and marked hyperplasia of the intrahepatic bile duct with significant expression of IL-17A. CsESPs-stimulated human cholangiocarcinoma cells (RBE) displayed elevated proliferation ability and produced higher level of IL-17A. Supernatant of CsESPs-RBE cells activated human hepatic stellate cells (LX-2) with upregulated production of α-SMA and collagen I. Knocking down of *IL-17A* in RBE cells by lentivirus attenuated the expression of α-SMA and collagen I in LX-2 cells incubated with supernatants of CsESPs-stimulated RBE cells. Under stimulation of CsESPs, the bile duct organoids became swelled with thickened and deformable walls and prominent IL-17A signals. These findings suggest that CsESPs may activate HSCs through a new pathway of stimulating biliary epithelium to produce IL-17A.

## Author summary

*C. sinensis* excretory and secretory products play critical roles in causing pathological changes of bile duct and periductal liver tissues, yet the mechanism of CsESPs triggering inflammation and fibrosis by breaking the barrier of biliary epithelium was unclear. In this study, we discovered the high levels of serum IL-17A in *C. sinensis* infected patients and mice, and significant IL-17A expression in biliary epithelium of mouse. RBE cells stimulated with CsESPs activated LX-2 cells to produce more α-SMA and collagen I through upregulating IL-17A expression, which was attenuated by knocking down of *IL-17A*. Bile duct organoid was firstly established and confirmed the production of IL-17A under stimulation with CsESPs.

## Introduction

*C. sinensis* is a vital food-borne parasite which mainly endemic in Asian countries including China, South Korea, Thailand, northern Vietnam and the Russian Far East, causing about 15 million people suffering from the infection worldwide [1,2]. As definitive hosts, mammals and human get infected by eating raw or undercooked freshwater fish carrying metacercariae. After the metacercariae excysted in the duodenum, the juveniles migrate along with bile chemotaxis into the intrahepatic bile duct and then grow up into adults there [3]. Living in the biliary tree, the worms cause serious damage to the bile duct and liver. Pathological changes of the bile duct such as marked dilatation of the duct, thickening of the ductal wall, hyperplasia of the biliary mucosa and periductal inflammation are triggered by mechanical obstruction, feeding and migratory activities of the worms. Hepatic fibrosis, hepatocirrhosis and even cholangiocarcinoma are common lethal consequences in the late stage. *C. sinensis* is classified as one of the group I biocarcinogens by the International Agency for Research on Cancer (IARC) and clonorchiasis is included in the control program of classified tropical diseases by WHO [4–6]. CsESPs play critical roles in stimulating periductal inflammation and hepatic fibrosis. As the first barrier between the worms and periductal liver tissues, bile duct epithelia suffer from the stimulation of CsESPs and play a pivotal role in triggering subsequent cascaded inflammatory responses which cause serious damage to liver tissues, such as hepatic fibrosis. Secretion of inflammatory factors by biliary epithelium is the beginning of subsequent recruitment of inflammatory cells and further pathological damages [7]. Studies showed that CsESPs could promote the proliferation of cholangiocarcinoma cells and induce the production of inflammatory factors such as IL-6 and TNF-α leading to the recruitment of neutrophils and monocytes, fostering a chronic inflammatory microenvironment. Continuous inflammatory stimulation further activates HSCs and drives excessive extracellular matrix (ECM) deposition which ultimately leads to hepatic tissue remodeling and periductal fibrosis [8–10].

The IL-17 family comprises six members including from IL-17A to IL-17 F with IL-17A the most extensively studied due to its abundance and biological activities. As the core component of the IL-17 family, IL-17A is primarily secreted by Th17 cells and also by natural killer T cells, CD8^+^ T cells, Kupffer cells and epithelial cells [11,12]. IL-17A plays important roles in anti-bacterial infections, inflammation, fibrotic diseases and autoimmune disorders with widely distributed receptors on various cells including eosinophils, keratinocytes, epithelial cells, HSCs and fibroblasts [13,14]. Recent studies have highlighted its significant functions in liver fibrosis. Tang *et al* found higher serum IL-17A level in liver fibrosis patients than in healthy individuals [15]. Besides, IL-17A was found to promote secretion of collagen I and α-SMA by enhancing HSCs’ reactivity to TGF-β and TNF-α. IL-17A also boosted HSCs chemokine secretion, for example, chemokine IL-8, CCL20 and MCP-1, to recruit and activate macrophages for pro-fibrotic cytokine release [16–18]. Moreover, IL-17A could exacerbate liver inflammation and fibrosis through synergizing with cytokines IL-22 and TNF-α [19]. So far, whether IL-17A plays a role in *C. sinensis* infection remains unclear. This study was designed to investigate the interaction between CsESPs and biliary epithelium and address the new-found mechanism that CsESPs may promote the liver fibrosis through biliary epithelial cells - IL-17A - HSCs axis.

## Methods

### Ethics statement

*C. sinensis* infected patient serum was collected from Guidong People's hospital and gifted by professor Xiaohong Peng of Guilin Medical University. Healthy participants serum was collected from physical examination department of Binzhou Medical University Hospital. Female BALB/c mice and male New Zealand White rabbits were purchased from Pengyue Bio China Inc and Xilingjiao Bio China Inc respectively. (Jinan, China) and kept under pathogen-free conditions according to Binzhou Medical University Hospital animal facility. Approval for human specimens and animal experiments was obtained from the Ethics Committee and Institutional Animal Care and Use Committee of Binzhou Medical University Hospital respectively (approval number 20221014–08 and KYLL-2025–045). This study was carried out in strict accordance with the recommendations in the Guide for the Care and Use of Laboratory Animals of the Ministry of Science and Technology of the People's Republic of China.

### Clinical characters of patients

Written informed consent was obtained from each patient or healthy participant included in the study. A total of 19 diagnosed *C. sinensis* infection patients and 19 healthy participants were recruited in this study. Inclusion criteria for this study were as follows: The healthy participants consisted of individuals undergoing health examinations who were excluded from *C. sinensis* infection, while the infection group comprised patients with just *C. sinensis* infection (positive for *C. sinensis* eggs in stool examination and positive for *C. sinensis* IgG antibodies). The exclusion criteria was that patients with viral hepatitis and non-viral liver diseases, including alcoholic liver disease, drug-induced liver injury, fatty liver disease, and metabolic liver diseases were excluded. Those with comorbid malignancies, diabetes, autoimmune diseases, AIDS or other diseases were also excluded. Additionally, patients aged below 18 or above 70 years old, as well as pregnant women were excluded [20,21].

### Parasite and animal infection

*Pseudorasbora parva* containing *C. sinensis* metacercariae were acquired from Wuhu City, Anhui Province, China. The metacercariae were isolated and collected by digesting the fish with an artificial gastric juice comprising pepsin (MP Biomedicals, Germany) and hydrochloric acid. All animals were maintained under controlled conditions (22 ± 2 °C, 12 h light/12 h dark cycle) with free access to standard laboratory feed and water. After one week of acclimatization, BALB/c mice (female, 7-week-old) were orally infected with 50 metacercariae by gavage and euthanized via cervical dislocation under anesthesia with tribromoethanol 4 weeks post-infection. Mice in control group received gavage of the same volume of sterilized saline. New Zealand White rabbits were infected intragastrically with total 600 metacercariae and were sacrificed under anesthesia with 3% sodium pentobarbital 6 weeks after infection. *C. sinensis* adult worms were recovered from the rabbit bile duct after the livers were removed. Animal experiments were conducted in strict accordance with the regulations for the administration of laboratory animals and every effort was made to minimize pain.

### Histological staining

Mouse liver tissues were fixed in 4% paraformaldehyde, embedded in paraffin and sectioned into 4 μm thick sections. For hematoxylin and eosin (HE) staining, after deparaffinization and rehydration, the sections were stained with hematoxylin for 5 min, rinsed and the stained with eosin for 2 min followed by dehydration, clearing, and mounting. For Masson’s trichrome staining, sections were processed according to the manufacturer's instructions (Solarbio, China). Briefly, after deparaffinization and rehydration, sections were stained with Weigert’s hematoxylin for 5–10 minutes, differentiated in 1% acid alcohol for 10–15 seconds and blued in Masson’s blueing solution. Subsequently, staining was carried out with Ponceau-Fuchsin solution followed by differentiation in phosphomolybdic acid and counterstaining with Aniline blue. After a brief fixation in 1% glacial acetic acid, sections were dehydrated, cleared in xylene and mounted with neutral balsam. All stained sections were observed and imaged under a light microscope.

### Immunohistochemical staining

Mouse liver ribbons were deparaffinized, rehydrated and placed on slides. Antigen retrieval was performed in a microwave using a low-pH antigen repair solution followed by cooling and washing in PBS. The slides were incubated with 0.3% H_2_O_2_ for 10 min at room temperature to block endogenous peroxidase and were blocked with goat serum at 37 °C for 30 min. After washing with PBS, the slides were incubated with rabbit anti-mouse IL-17A monoclonal antibody (1:5000, Bioss, China) overnight at 4 °C. After washing, the slides were subsequently incubated with horseradish peroxidase-conjugated goat anti-rabbit IgG (undiluted, ZSGB-BIO, China) for 20 min at 37 °C. The target IL-17A was visualized by the substrate of diaminobenzidine with a chromogenic development time of 2 min. The target signals were analyzed statistically by GraphPad Prism 8.0.

### Enzyme-linked immunosorbent assay (ELISA) for IL-17A

Mouse blood samples were collected 4 weeks after infection. Sera were isolated by centrifuge and used to determine the concentration of IL-17A using commercially available ELISA kits according to the manufacturer's instructions (Elabscience, China). The procedure was as follows: human or mouse serum was aliquoted into 96-well plate (human, 100 μL/well; mouse, 25 μL/well) and incubated at 37°C for 90 min. Then, the plate was incubated with biotinylated detection antibody (1:100) at 37°C for 60 min. After washing three times with PBS containing 0.1% Tween-20, the plate was incubated with HRP conjugate at 37°C for 30 min and washed five times repeatedly. The samples were treated with the substrate reagent and the reaction was terminated with stop solution. Optical density was measured at 450 nm using a microplate reader and all measurements were carried out in triplicates.

### *C. sinensis* ESPs (CsESPs) preparation

The isolated adult worms were washed with sterilized PBS three times and cultured in PBS with 1% penicillin/streptomycin (Beyotine, China) with 5% CO_2_ at 37˚C for 24 h. The supernatant was collected and used as CsESPs after centrifuge to remove the eggs, adult worm lysates and other substances, and kept in -80℃ freezer until use. The protein concentration was measured using BCA protein Assay (SparkJade, China).

### Cell culture and stimulation

RBE cells (Procell, China) were cultured in RPMI-1640 containing 10% fetal bovine serum (FBS) (Gibco, USA) and 1% penicillin/streptomycin (Beyotime, China) with 5% CO_2_ at 37°C. The cells were seeded in six-well plate at 2 × 10^5^ cells/well and stimulated with 400 ng/mL of CsESPs or the same volume of PBS for 24 h after being starved with no-serum culture medium for 6 h. The supernatants were collected, centrifuged, and the concentration was measured by BCA assay. LX-2 cells (Procell, China) were cultured in DMEM medium containing 10% FBS and 1% penicillin/streptomycin with 5% CO_2_ at 37°C and stimulated with RBE cells cultured supernatants at a final concentration of 1 µg/mL for 24 h. The supernatant was collected for further detection of the production of collagen I and α-SMA.

### Knock down of IL-17A from RBE Cells by lentivirus transfection

The RBE cells in the logarithmic growth stage were divided into three groups (blank, control lentivirus and shIL-17A) and seeded into 6-well plate. Upon reaching 60% cell density, 2 μL of control lentivirus (expressing GFP, GFP-control) or IL-17A-targeting lentivirus (expressing GFP, GFP-shIL-17A) solution (Haixing Biosciences) was introduced into the cell medium respectively. The infection rate was visualized by detecting the intensity of green fluorescence of GFP 72 h after infection. The cells with shIL-17A were screened by the addition of puromycin at a concentration of 2 μg/mL. qRT-PCR and western blotting were performed to validate the knockdown efficiency. Cells in blank group received no special treatment. All three groups of cells were stimulated with CsESPs or PBS as above.

### Cell proliferation assay (CCK-8)

The Cell Counting Kit-8 (CCK-8) assay (Elabscience, China) was performed to assess cell proliferation and activity according to the manufacturer's instructions. Briefly, the RBE cells suspension was adjusted to 1 × 10^5^ cells/mL and 100 µL of the cell suspension was cultured in each well of 96-well plate with 400 ng/mL CsESPs or equal volume of PBS. All cells were cultured with CCK-8 solution addition at different time points of 0 h, 12 h, 24 h, 48 h, and 72 h respectively. The absorbance of each well at 450 nm was measured using an enzyme-linked immunosorbent detector.

### Quantitative analysis of target genes (qRT-PCR)

The total RNA was extracted from RBE or LX-2 cells using TRIzol reagent (Solarbio, Beijing, China) according to the manufacturer's instructions. Briefly, RBE cells were serum-starved for 6 h and then stimulated with 400 ng/mL CsESPs for 24 h. LX-2 cells were treated for 24 h with culture supernatants from RBE cells at a final concentration of 1 µg/mL as described in the cell culture and stimulation section. The first-strand cDNA was synthesized using Power cDNA Synthesis Kit (SparkJade, China) according to the manufacturer's protocol. Oligo 6 program was applied to design the primers and the gene of glyceraldehyde-3-phosphate dehydrogenase was employed as a reference gene (listed in Table 1). Thermal cycle was performed using CFX96 (BIO-RAD, USA) as follow: the reaction mix was heated to 95℃ for 3 min firstly followed by 40 cycles of 94℃ for 20 sec, 58℃ for 20 sec and 72℃ for 30 sec. Relative transcriptional level was calculated using 2-^△△^^Ct^ method.

**Table 1:** Primer sequences for quantitative real-time PCR.

Gene	Forward	Reverse
*GAPDH*	5’-GGAGCGAGATCCCTCCAAAAT-3’	5’-GGCTGTTGTCATACTTCTCATGG-3’
*IL-17A*	5’-CCTCATTGGTGTCACTGCTACTG-3’	5’-TTGTCCTCAGAATTTGGGCATCC-3’
*α-SMA*	5’-TTACGAGTTGCCTGATGG-3’	5’-TGCTGTTGTAGGTGGTTTC-3’
*Collagen-I*	5’-GTGCGATGACGTGATCTGTGA-3’	5’-CGGTGGTTTCTTGGTCGGT-3’

### Western blot

Cells and tissues were lysed using RIPA buffer with protease and phosphatase inhibitors (Solarbio, China) and then centrifuged at 12,000 × g for 15 min at 4 °C to remove debris. The supernatants were collected and the protein concentration was measured via BCA assay. The proteins were separated by 12% SDS-PAGE and then transferred to PVDF membranes. After blocked with 5% skimmed milk for 2 h at room temperature, the membranes were incubated with rabbit anti-human IL-17A (1:500, Bioss, China), rabbit anti-mouse IL-17A (1:500, Bioss, China), rabbit anti-human α-SMA (1:1000, Bioss, China), rabbit anti-human collagen I(1:1000, Bioss, China), β-actin, and GAPDH monoclonal antibodies (1:10000, Abcam, USA) respectively at 4 ℃ overnight. Then, the membranes were washed and incubated with HRP-conjugated goat anti-rabbit IgG antibodies (1:10000, BOSTER, China) for 60 min at room temperature. Blots were visualized using an ECL kit (BIOVIGEN, China).

### The construction of mouse bile duct organoid

Normal mouse liver was isolated and processed as follows: after washing with Dulbecco’s Phosphate Buffer (DPBS) (Procell, PB180329), the liver tissue was cut into small pieces (0.5-1 mm) using sterile scalpels and digested in MasterAim tissue enzymatic solution I (AIMINGMED, 10100047) at 37 °C for 30 min with intermittent shaking. The digestion was terminated by adding DPBS followed by centrifugation for 5 min and the supernatant was discarded. After tissue digestion solution II (AIMINGMED, 10100048) was added, the mixture was incubated at 37 °C for 15 min and the reaction was stopped by DPBS. The sample was centrifuged at 300 g for 5 min and the supernatant was removed. The cell pellet was re-suspended in DPBS and filtered through a 100-μm diameter strainer which was pre-moistened with anti-adhesion solution. The pass through was centrifuged and the supernatant was discarded. The cell pellets were mixed with matrix gel and plated into 24-well plates at a density of 1 × 10^5^ cells/well. The plates were incubated with organoid medium (AIMINGMED, 10100400) with 5% CO₂ at 37 °C. The morphology of mouse bile duct organoids was observed under a microscope and the validity was identified by the expression of cytokeratin 19 (CK19).

### Immunofluorescence in mouse bile duct organoids

Upon subculturing of the organoids, the experimental group was incubated with CsESPs at a final concentration of 5 μg/mL, whereas the control group received an equal volume of PBS. Three days later, organoids were washed with DPBS-PS and fixed in 4% paraformaldehyde for 10 min at room temperature. Antigen retrieval was performed by adding citrate antigen retrieval solution (pH 6.0) and heating in a boiling water bath for 20 min. The organoids were then permeabilized with 0.2% Triton X-100 for 1 h and blocked with blocking solution for 2 h at room temperature. Subsequently, the organoids were incubated with rabbit anti-mouse CK19 or IL-17A antibody overnight at 4°C and then with FITC or TRITC conjugated goat anti-rabbit IgG antibody respectively. After washing three times with DPBS-PS, the nuclei were stained with dihydrochloride (DAPI) for 10 min in the dark and the samples were imaged using a confocal microscope.

### Statistical analysis

All data were analyzed using GraphPad Prism 8.0 or ImageJ and were presented as the mean ±standard error of the means (SEM). Comparisons between two groups were evaluated using Student's t test. Differences among the groups were tested using a one-way ANOVA. All results were considered as statistical significance when *P* < 0.05.

## Results

### The expression level of IL-17A was increased in the serum of patients infected with *C. sinensis*

**Fig 1:**
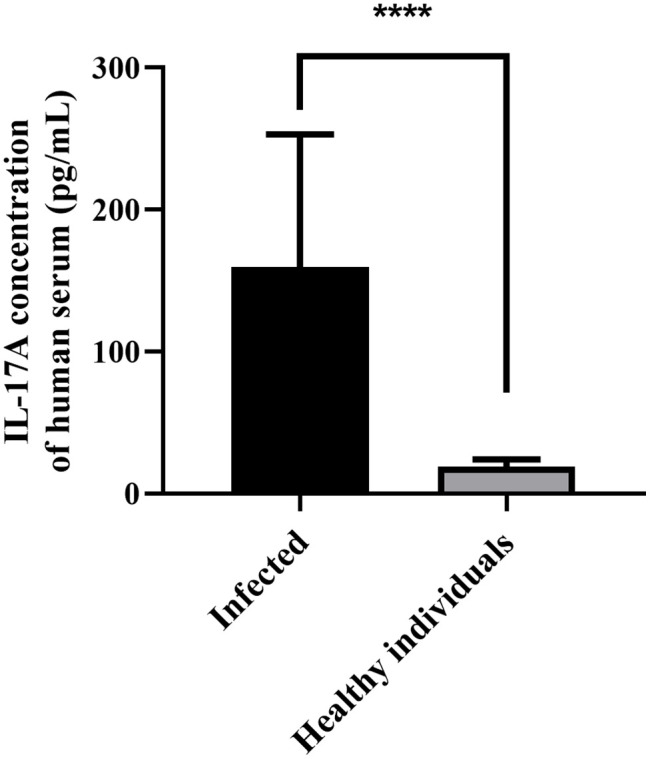
Expression level of IL-17A in human serum. The IL-17A level was significantly higher in serum of patients infected with *C. sinensis* than that of healthy individuals. * *P* < 0.05, ** *P* < 0.01, *** *P* < 0.001, **** *P* < 0.0001.

In order to investigate the relationship between *C. sinensis* infection and IL-17A expression, serum samples from 19 infected patients and 19 healthy individuals were collected. IL-17A levels were measured using ELISA. The result showed that the IL-17A level was significantly higher in infected patients than in healthy individuals (Fig 1, *P* < 0.0001).

### Effects of *C. sinensis* infection on liver fibrosis and IL-17A expression in mice

**Fig 2:**
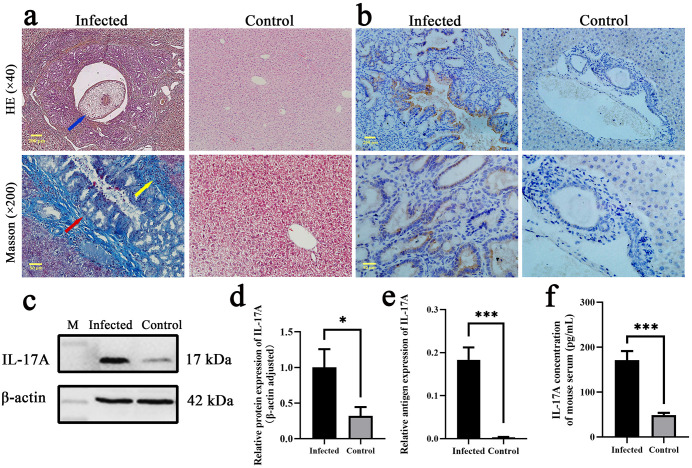
Effects of *C. sinensis* infection on mouse liver and IL-17A expression. **a** HE and Masson’s trichrome staining of mouse liver, blue arrow indicates *C. sinensis* adult worm in the bile duct lumen; red arrow indicates collagen deposition and biliary hyperplasia; yellow arrow indicates inflammatory cells infiltration. **b** Immunohistochemical staining showed localization of IL-17A in the intrahepatic biliary epithelium of mouse infected with *C. sinensis* (brown signals). **c, d** Western blot revealed significantly elevated protein level of IL-17A in the liver of *C. sinensis*-infected mouse. **e** Quantitative analysis of IL-17A-positive signals in immunohistochemical staining. **f** IL-17A levels in *C. sinensis*-infected and control mouse serum. * *P* < 0.05, ** *P* < 0.01, *** *P* < 0.001, **** *P* < 0.0001.

HE and Masson’s trichrome staining were applied to investigate the pathological changes and fibrosis of mouse liver caused by *C. sinensis* infection. The results revealed that, compared with mice in the control group, liver tissues of *C. sinensis* infected mice exhibited significant inflammation with conspicuous inflammatory cells infiltration around the bile ducts and extensive collagen fiber deposition 4 weeks after infection (Fig 2a). To explore the influence of *C. sinensis* infection on IL-17A expression, the serum IL-17A was measured by ELISA and displayed significantly higher level in the infected group than the control (Fig 2f, *P* < 0.001). Consistently, western blot analysis showed that total IL-17A protein level in the liver of infected mice was also significantly higher than that of the control (Fig 2c and 2d, *P* < 0.05). Immunohistochemical staining indicated that IL-17A was mainly expressed in the intrahepatic bile duct of infected mouse and displayed significantly higher signal level than that of control mouse (Fig 2b and 2e, *P* < 0.001).

### CsESPs activated HSCs by stimulating biliary epithelial cells

**Fig 3:**
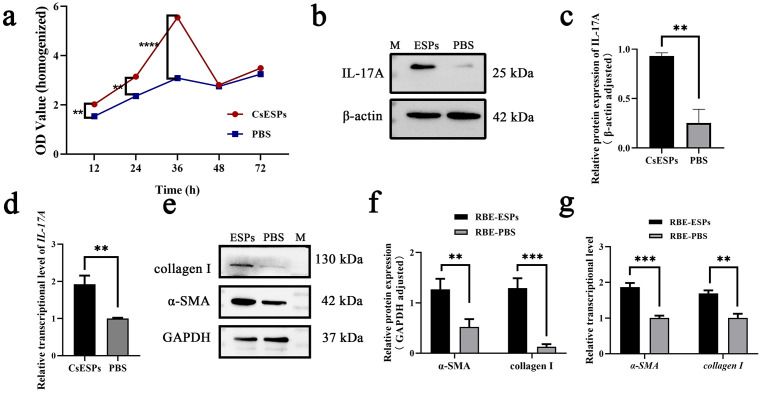
CsESPs activated HSCs through stimulating biliary epithelial cells to produce IL-17A. **a** Effect of CsESPs on the proliferation of RBE cells. **b, c** The protein levels of IL-17A in RBE cells were significantly elevated by CsESPs stimulation. **d** The transcriptional levels of *IL-17A* in RBE cells were significantly elevated by CsESPs stimulation. **e, f** The protein levels of α-SMA and collagen I in LX-2 cells were significantly elevated after treatment with CsESPs-RBE supernatants. **g** The transcriptional levels of *α-SMA* and *collagen I* were obviously elevated in LX-2 cells treated with CsESPs-RBE supernatants. * *P* < 0.05, ** *P* < 0.01, *** *P* < 0.001, **** *P* < 0.0001.

Since the RBE cell line originated from intrahepatic biliary epithelium, they were used as the cell model of biliary epithelial cells in this study. CCK8 was performed to access the influence of CsESPs on RBE cells. The results showed that the proliferation of RBE cells were significantly accelerated at 12 h, 24 h, and 36 h after stimulation by CsESPs compared with the control group (Fig 3a, *P* < 0.01). qRT-PCR and western blot analysis displayed that IL-17A expression was significantly increased in CsESPs-treated RBE cells at both the transcriptional and protein levels (Fig 3b, 3c and 3d *P* < 0.01). To explore whether CsESPs promote hepatic fibrosis through stimulating biliary epithelium, the culture supernatants of CsESPs or PBS-treated RBE cells were added to the LX-2 cells culture system. The results demonstrated that the expression of α-SMA and collagen I was significantly increased in LX-2 cells treated with supernatants of CsESPs-RBE cells at both the transcriptional and protein levels (Fig 3e, 3f and 3g, *P* < 0.01).

### Knocking down of IL-17A in RBE cells attenuated the activation of HSCs

**Fig 4:**
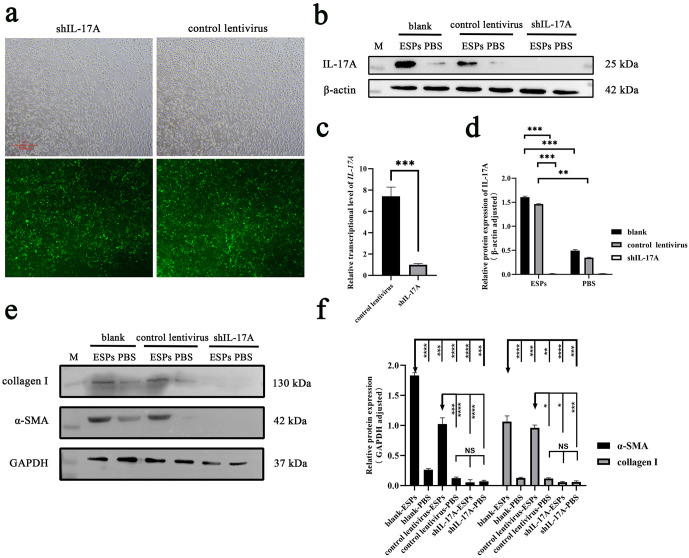
Knockdown of IL-17A in RBE cells by shRNA inhibited the activation of HSCs. **a** Infection efficiency of lentivirus to RBE cells by GFP fluorescence assay. **b, d** CsESPs induced IL-17A expression in RBE cells of blank and control lentivirus groups but not in shIL-17A group. Blank (RBE cells without lentiviral transduction), control lentivirus (RBE cells transduced with control lentivirus), shIL-17A (RBE cells transduced with IL-17A-targeting lentivirus). **c** qRT-PCR performed to assess the knockdown efficiency of *IL-17A* in RBE cells infected with lentiviruses. **e, f** Knockdown of IL-17A reduced the expressions of collagen I and α-SMA in LX-2 cells treated with RBE cell culture supernatant. * *P* < 0.05, ** *P* < 0.01, *** *P* < 0.001, **** *P* < 0.0001.

To further explore whether CsESPs activate HSCs through stimulating the secretion of IL-17A by biliary epithelium, lentiviruses (shIL-17A) were used to infect RBE cells to knock down IL-17A. 72 h after infection, the infection efficiency of lentivirus in RBE cells was about 80% visualized by green fluorescent protein under a fluorescent microscope (GFP, Fig 4a). qRT-PCR and western blot analysis showed no IL-17A expression at both the transcriptional and protein levels in shIL-17A infected RBE cells indicating the high knocking down efficiency (*P* < 0.01, Fig 4b and 4c). After being incubated with CsESPs, RBE cells in the blank and control lentivirus-infected groups displayed higher expression level of IL-17A compared with shIL-17A infected cells which showed no target band (*P* < 0.01, Fig 4b and 4d). The supernatants of RBE cells were added to the LX-2 cells culture system which simulated the stimulation of HSCs by biliary epithelium secretions *in vivo*. The results showed that the expression of α-SMA and collagen I were significantly lower in shIL-17A-RBE supernatant-stimulated LX-2 cells than the other two groups demonstrating that CsESPs could promote the activation of HSCs through irritating the biliary epithelium to secrete IL-17A (Fig 4e and 4f).

### CsESPs promoted the hyperplasia and IL-17A expression of mouse bile duct organoids

**Fig 5:**
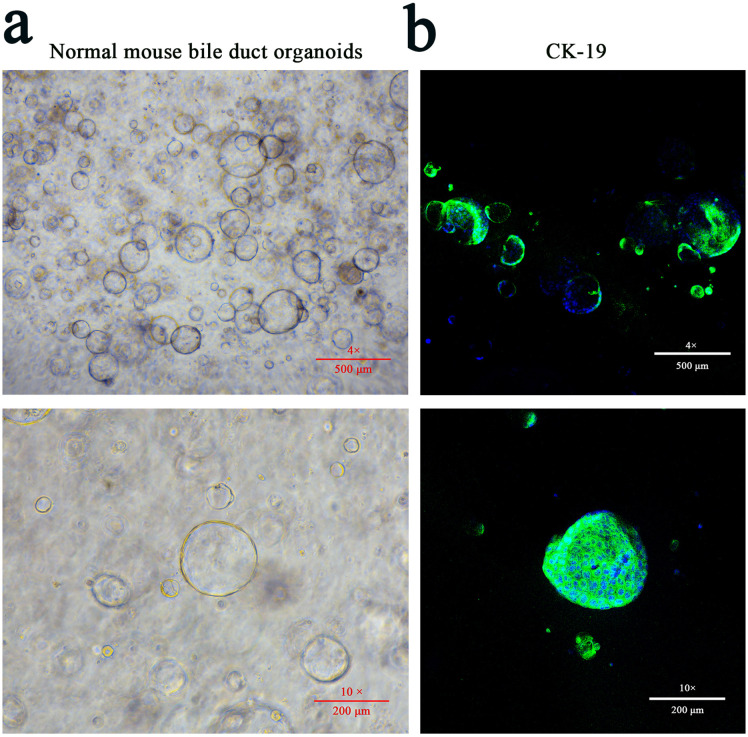
Morphology of normal mouse bile duct organoids. **a** Morphology of primary mouse bile duct organoids 10 days post-seeding. **b** Immunofluorescence detection of the expression of CK-19 in the biliary epithelium (CK-19, fluoresces green; DAPI, fluoresces blue).

**Fig 6:**
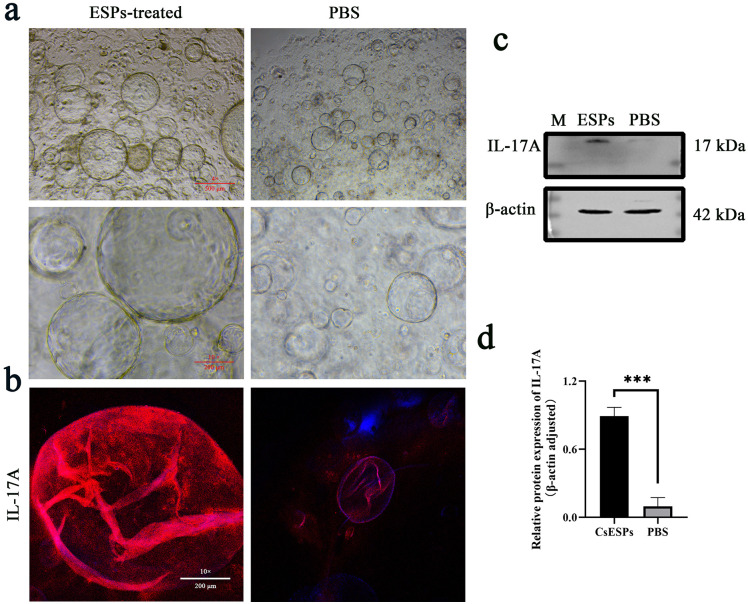
CsESPs induced IL-17A expression in mouse bile duct organoids. **a** Morphology of mouse bile duct organoids after treatment with CsESPs or PBS. **b** The expression of IL-17A was detected by immunofluorescence (IL-17A, fluoresces red; DAPI, fluoresces blue). **c, d** The protein level of IL-17A was analyzed by western blot. * *P* < 0.05, ** *P* < 0.01, *** *P* < 0.001, **** *P* < 0.0001.

To further simulate the real influence of CsESPs on biliary epithelium *in vivo* and avoid the interference of other IL-17A-producing cells in liver tissue, such as Th17 cells, a bile duct organoid model of BALB/c mouse was established in this study. The primary bile duct cells aggregated into cell clusters firstly and then formed circular confluent layers. At about 10 days, swollen circular structures were observed (Fig 5a). Immunofluorescence staining showed the expression of specific biomarker CK-19 in the organoid indicating successful construction of bile duct organoids (Fig 5b). After stimulation with CsESPs, the organoids exhibited enlarged and swollen morphology with thickened and deformable bile duct walls compared with those of PBS-stimulated. (Fig 6a). Immunofluorescence and western blot analysis revealed that IL-17A expression in the CsESP-stimulated organoids was significantly increased (Fig 6b, 6c and 6d).

## Discussion

*C. sinensis* is a worm that primarily induces pathological changes of bile ducts and surrounding liver tissues, such as bile duct hyperplasia, inflammation, liver fibrosis and even cholangiocarcinoma. The complex pathological process is driven by the combined effects of mechanical damage from the worm, chemical stimulation from CsESPs and the following remodeling of the immune microenvironment. These factors together drive the progression from inflammation to fibrosis and even cholangiocarcinoma [3]. CsESPs, containing immunogenic and chemically toxic proteins and bioactive substances, play critical roles in inducing the above pathological changes. As the first barrier between the worm and liver tissue, biliary epithelium suffers from the stimulation of CsESPs firstly and play the role of a switch for the following liver damage. Under the stimulation of CsESPs, the bile duct displays abnormal proliferation and release inflammatory cytokines which will cause cascades of inflammation and further liver injury [22,23]. In the present study, thickened bile duct and periductal inflammation were observed in the periductal tissue of mice infected with *C. sinensis*. CCK-8 assay also showed that CsESPs promoted RBE cells proliferation confirming the influence of CsESPs on biliary epithelium. In previous study, IL-6 and TGF-β was upregulated by CsESPs in H69 cells suggesting secretion of inflammatory factors by biliary epithelium [24,25].

IL-17A is a pleiotropic pro-inflammatory cytokine and plays key roles in regulating inflammatory responses. Its high-expression is closely related to various inflammatory diseases and tumor progression, and is often used to monitor tumor growth, metastasis, and angiogenesis [13,26]. In recent years, IL-17A was found to promote or trigger hepatic fibrosis by inducing the release of pro-inflammatory mediators such as IL-6, TNF-α, IL-22, and CCR6 [27,28]. Kartasheva *et al* analyzed the liver tissues and blood samples from patients with various stages of liver fibrosis and found that the expression of IL-17A was significantly increased in the early stages of fibrosis [29]. As we know, HSCs activation is the core event of hepatic fibrosis. It is reported that IL-17A upregulated the expression of α-SMA in HSCs by enhancing HSCs’ reactivity to TGF-β through the JNK pathway [30]. Besides, IL-17A also played a role in upregulating and stabilizing TGF-β receptor causing the increase of SMAD 2/3 signaling, the most important signal for HSCs activation [31]. Some study revealed that IL-17A could even directly induce fibroblast activation protein-α (FAP) production in HSCs through the STAT3 pathway [32]. In the present study, the expression level of IL-17A significantly increased in the serum of *C. sinensis* infected patients and mice indicating that it may play important role in liver damage or fibrosis. Yan C *et al* found that IL-17A increased from 14 d post *C. sinensis* infection while the frequency of Th17 cells increased on day 56 indicating that IL-17A was produced not by Th17 cells in the early infection stage [33]. Although Th17 cells are the major source of IL-17A, the epithelial cells can also produce IL-17A. For example, nasal epithelial cells could promote the formation of nasal polyps through autocrine IL-17A [34]. In our study, the immunohistochemical staining dionisplayed that IL-17A was mainly located in the biliary epithelium of mice infected with *C. sinensis* 4 weeks post infection. Consistently, the biliary epithelium cell line (RBE cells) also produced IL-17A after stimulation with CsESPs confirming the secretion of IL-17A by biliary epithelium. In order to clarify the roles of IL-17A produced by biliary epithelium in HSCs stimulation, we cultured LX-2 cells with supernatants of CsESPs-stimulated RBE cells and found that the production of collagen I and α-SMA were boosted significantly suggesting that CsESPs could activate HSCs through inducing the secretion by biliary epithelium. Next, we found that the expression of collagen I and α-SMA did not increase after IL-17A was knocked down indicating the direct activation effect of IL-17A to HSCs. These results collectively identify a novel mechanism contributing to *C. sinensis*-associated hepatic fibrosis which may cooperates with other CsESPs-induced factors to drive the progression of periductal fibrogenesis.

However, it is noteworthy that in the present study, knockdown of IL-17A in RBE cells almost completely abolished the induction of α-SMA and collagen I in LX-2 cells by CsESPs-stimulated supernatants, despite the fact that liver fibrosis is known to be a multifactorial process involving multiple cytokines and cell types. The possible reason may be that IL-17A may act as a critical upstream amplifier which promote other potential pro-fibrotic factors (e.g., IL-6, TGF-β) to trigger HSC activation. Additionally, IL-17A has been reported to synergize with other cytokines such as TNF-α and IL-22 to promote fibrosis [27,28], suggesting that its depletion may disrupt a cooperative network essential for HSC activation. Nevertheless, the current cell model could not simulate the complicated microenvironment and network *in vivo*, including infiltrating immune cells and stromal components, which may contribute to various pro-fibrotic signals. Therefore, the apparent dominance of IL-17A in this experimental setting does not exclude the involvement of other factors in vivo, but rather highlights its indispensable role in initiating the pro-fibrotic cascade triggered by CsESPs.

Organoid is a kind of three-dimensional cultures that mimic the histology, pathology, structure and genetics of tissues or organs *in vivo* offering a potential alternative to cell cultures, xenografts and animal models. Compared with traditional cell cultures, organoids imitate cell-cell, cell-ECM interactions and biological processes more accurately [35]. Due to resembling the body's tissue environment and be capable of exposure to biomolecules such as hormones, chemokines, nutrients and waste, organoids can simulate *in vivo* cellular processes of migration, proliferation, differentiation, survival and morphogenesis in various tissues and organs [34]. Until now, no organoids model was used in the study of *C. sinensis* infection. In the present study, a bile duct organoid model of BALB/c mice was successfully established with the morphology and CK-19 expression to investigate the influence of CsESPs on the proliferation and IL-17A secretion of biliary epithelium. After stimulation with CsESPs, the organoids became enlarged and swollen with thickened and deformable bile duct walls similar with the pathological changes *in vivo*. Western blot and immunofluorescence revealed the upregulated expression of IL-17A which was consistent with the results of cell experiment and immunohistochemical staining of mouse live tissues further confirming the production of IL-17A by biliary epithelium under the stimulation of CsESPs.

## Conclusions

CsESPs play a key role in trigging the inflammation and the following hepatic fibrosis. The mechanism of CsESPs breaking the barrier of bile duct and provoking further pathological changes remains unclear. In the present study, IL-17A was found highly expressed in *C. sinensis* infected patient and mouse serum and in mouse biliary epithelium. CsESPs stimulated bile duct epithelial cells to secrete high level of IL-17A which further activated HSCs to produce more collagen I and α-SMA. In summary, this study indicates that CsESPs may promote liver fibrosis by stimulating biliary epithelial cells to secrete IL-17A which will subsequently trigger peridictal inflammation and activation of HSCs. This study provides a new sight for the mechanism of liver fibrosis caused by *C. sinensis* and is helpful to find new therapeutic targets.
